# Developmental stage–dependent neurotoxicity of sevoflurane: evidence from brain organoids

**DOI:** 10.3389/fncel.2026.1774547

**Published:** 2026-03-09

**Authors:** Niqi Chen, Yanting Zhang, Yeru Chen, Gang Chen

**Affiliations:** 1 Department of Anesthesiology, Sir Run Run Shaw Hospital, School of Medicine, Zhejiang University, Hangzhou, Zhejiang, China; 2 Provincial Key Laboratory of Precise Diagnosis and Treatment of Abdominal Infection, Sir Run Run Shaw Hospital, School of Medicine, Zhejiang University, Hangzhou, Zhejiang, China

**Keywords:** brain organoids, developmental neurotoxicity, ferroptosis, mitochondrial dysfunction, neural progenitor cells, neuroinflammation, oxidative stress, sevoflurane

## Abstract

Sevoflurane is widely used in pediatric anesthesia because it allows rapid induction and recovery, yet its use during vulnerable periods of brain development has raised concerns about long-term neurocognitive effects. Experimental data indicate that sevoflurane engages multiple interacting pathways, including mitochondrial dysfunction, oxidative and iron-dependent injury, and immune-mediated synaptic and inflammatory responses, whose net impact depends on dose, timing, and exposure duration. Human brain organoids have meanwhile emerged as human-based three-dimensional models that reproduce key aspects of cortical and midbrain development and permit clinically relevant exposure paradigms to be tested *in vitro*. In both cortical-like and midbrain-like organoids, sevoflurane narrows and disorganizes progenitor zones, disrupts interkinetic nuclear migration, reduces apical mitoses, and accelerates neuronal or dopaminergic differentiation, with more pronounced changes after more intense exposure. These architectural alterations are accompanied by molecular and electrophysiological signatures of mitochondrial stress, iron dysregulation, and inflammatory activation, forming multidimensional “injury fingerprints” that parallel clinical observations that single, brief anesthetics rarely cause overt global decline, whereas repeated or prolonged anesthetics are associated with subtler, domain-specific deficits. This review synthesizes current evidence on the developmental stage–dependent effects of sevoflurane and highlights how brain organoids are being used to dissect underlying cellular and molecular mechanisms. It also discusses key limitations of current organoid systems and considers how more mature, vascularized, and microglia-containing models, integrated with perioperative cohorts and targeted interventions, may eventually inform exposure-aware anesthetic planning without delaying necessary surgery. Overall, this review advances a developmental stage–aware, organoid-centered conceptual framework that integrates animal and human-based evidence to better understand and stratify the risk of sevoflurane-induced neurotoxicity.

## 1 Introduction

In clinical practice, numerous infants and young children receive general anesthesia during critical periods of early development due to surgical procedures or imaging examinations (Patel and Goa, 1996; Warner et al., 2018; Xiao et al., 2022). Among inhaled anesthetics, sevoflurane is widely preferred owing to its favorable pharmacokinetic profile, characterized by rapid induction and emergence, thereby establishing it as a cornerstone of pediatric anesthetic management (Patel and Goa, 1996). Concurrently, a growing body of multiple animal datasets and human cohort analyses indicates that the developing brain is particularly vulnerable to anesthetic-induced disruptions (Sun M. et al., 2021; Warner et al., 2018; Xiao et al., 2022). Repeated or prolonged exposure to sevoflurane has been shown to interfere with key neurodevelopmental processes—including apoptotic signaling in neurons, glial maturation, synaptogenesis, and neuroendocrine crosstalk—and is associated with long-term cognitive deficits and socio-behavioral impairments (Liang et al., 2023; Shen et al., 2013; Sun M. et al., 2021; Zhao et al., 2022). These mechanistic pathways are consistently documented in comprehensive reviews on sevoflurane-induced developmental neurotoxicity, underscoring the imperative for rigorous assessment of dosage, timing, and duration of administration during windows of heightened susceptibility (Sun M. et al., 2021).

Beyond apoptosis-centered models, dysregulation of iron metabolism and redox homeostasis has emerged as one parallel axis contributing to neuronal vulnerability during neurodevelopment rather than replacing apoptosis (Sun M. et al., 2021). Sevoflurane and related volatile anesthetics can disturb cerebral iron handling and promote lipid peroxidation consistent with ferroptotic injury, and this has been linked—across neonatal and aging models—to later cognitive and memory impairments in a dose- and timing-dependent manner (Kang et al., 2023; Miao et al., 2024; Zeng et al., 2024). This pattern suggests that, in addition to limiting exposure, it may be possible to mitigate injury by modulating iron availability or reinforcing core ferroptosis-regulatory pathways (e.g., GPX4/SLC7A11), providing a mechanistically grounded set of targets for preclinical testing in human brain organoids (Miao et al., 2024; Sun M. et al., 2021).

The choice of model fundamentally shapes the scope and validity of the conclusions that can be drawn (Qian et al., 2019; Trujillo and Muotri, 2018). Two-dimensional cultures fail to recapitulate native tissue architecture, and inherent species differences impede the translational relevance of findings from rodent models to humans (Qian et al., 2019; Trujillo and Muotri, 2018).

Human brain organoids—self-organized three-dimensional structures derived from pluripotent stem cells—more accurately recapitulate human-specific developmental timelines, cellular diversity, and spatial organization, thereby enabling mechanistic investigation and preclinical evaluation within a fully human genetic context (Li et al., 2023; Sun N. et al., 2021; Trujillo and Muotri, 2018). Recent comprehensive reviews systematically summarize the strengths and limitations of human brain organoids and identify specific strategies—such as single-cell profiling, genome editing, incorporation of vascularization and immune components, and functional activity readouts—to enhance their experimental fidelity and reproducibility (Li et al., 2023; Qian et al., 2019; Sun N. et al., 2021), thereby helping to shorten the translational gap between laboratory findings and perioperative clinical practice (Cao, 2022; Li et al., 2023).

Taken together, the central role of sevoflurane in pediatric anesthesia and the heightened developmental sensitivity of the brain create a clinical paradox: the very pharmacologic properties that enable rapid and well-tolerated anesthesia in early life also coincide with critical periods of neurodevelopment, potentially disrupting vulnerable neural programs (Patel and Goa, 1996; Sun M. et al., 2021). Building on the above background, the following section transitions from contextual foundations to clinical application by delineating the use of sevoflurane across different age groups and procedural settings, and subsequently aligning these clinical scenarios with putative neuroinjury mechanisms—ranging from apoptosis-associated circuit dysfunction to iron dyshomeostasis and ferroptosis—thereby enabling a coordinated discussion of exposure parameters (dose, duration, timing) in relation to specific biological vulnerabilities (Kang et al., 2023; Miao et al., 2024; Sun M. et al., 2021). In parallel, we will highlight how human brain organoid models complement animal studies and two-dimensional cell systems by offering a human-relevant experimental platform for evaluating exposure paradigms and candidate protective interventions, thereby strengthening the connection between real-world clinical practice and mechanism-based safety assessment (Cao, 2022; Li et al., 2023; Sun N. et al., 2021).

## 2 Effects of sevoflurane on the nervous system at different developmental stages

Across development, sevoflurane’s effects on the nervous system are neither uniform nor static but highly stage-dependent—a pattern that aligns closely with the multi-node mechanisms outlined earlier (mitochondria–apoptosis, immune–synapse, iron–lipid/ferroptosis, and pyroptosis) and reinforces the clinical importance of exposure parameters such as dose, duration, and timing (Sun M. et al., 2021). Evidence from prenatal to aging models indicates that the younger the brain and the greater the synaptic and circuit activity, the narrower the safety margin; in contrast, adult and aged brains exhibit distinct, and sometimes bidirectional, responses that depend on baseline inflammatory tone and metabolic reserves (Sun M. et al., 2021; Sun et al., 2024).

Fetal/prenatal: Studies on maternal anesthesia demonstrate that sevoflurane crosses the placental barrier and disrupts fetal neurodevelopment (Song et al., 2017; Wan et al., 2022; Zheng et al., 2013). Exposure during mid-gestation leads to neuronal loss, deficits in dendritic spines, and impairments in learning and memory in offspring (Song et al., 2017; Wan et al., 2022; Zheng et al., 2013). Mechanistically, these effects involve CB1R-CDK5-p-tau signaling as well as canonical apoptotic and inflammatory cascades (Wan et al., 2022; Zheng et al., 2013). Independent cohorts have reported fetal and neonatal neurotoxicity following maternal exposure, further underscoring that the timing of exposure within gestation is a critical determinant of neurodevelopmental risk (Zheng et al., 2013).

Neonatal and early postnatal: During the period of rapid synaptogenesis, exposure history plays a critical role (Qiu et al., 2016; Zhao et al., 2022). Brief, single exposures (on the order of tens of minutes) may induce transient or subtle alterations in dendritic spine density or neuronal physiology, with limited long-term behavioral consequences in certain experimental paradigms (Qiu et al., 2016, 2024). In contrast, repeated or prolonged anesthesia suppresses hippocampal long-term potentiation (LTP), exacerbates neuroinflammation, and leads to age-dependent impairments in learning and memory (Shen et al., 2013; Zhao et al., 2022). Multiple independent datasets point to a tau-centered vulnerability in neonates: sequential phosphorylation at Ser262, Ser202, and Thr205 correlates with cognitive decline following multi-day exposure, whereas single exposures produce milder effects (Liang et al., 2023). Parallel studies demonstrate that sevoflurane rapidly increases dendritic spine density in the medial prefrontal cortex during synaptogenesis-changes that may occur independently of apoptosis but can still reconfigure circuit assembly (Briner et al., 2010). Long-term outcomes include persistent alterations in dendritic complexity extending into adolescence after a single exposure at postnatal day 7, indicating a durable impact on structural maturation (Useinovic et al., 2023). These phenotypes are consistent with the mechanisms outlined earlier, implicating microglial complement-mediated synaptic pruning, mitochondrial stress, and redox-lipid imbalance as cooperating drivers of neurodevelopmental disruption (Kang et al., 2023; Sun M. et al., 2021; Wang et al., 2024).

Childhood: In children, intra-anesthetic EEG signatures undergo dynamic changes with brain maturation: δ-wave predominance in early infancy transitions to emerging θ/α rhythms by approximately 4–6 months of age, followed by progressive frontal α synchrony during the latter part of the first year. These developmental trajectories reflect thalamocortical network maturation and support age-appropriate anesthetic management, as identical end-tidal concentrations may elicit distinct network states across different developmental stages (Cornelissen et al., 2018).

Adolescence and adulthood: Early-life exposure can lead to consequences that emerge during adolescence, such as increased dendritic complexity or atypical spine density (Useinovic et al., 2023). In contrast, acute sevoflurane administration in young mice induces neuronal hyperactivation and transient hyperlocomotion—a phenotype that is not readily observed in mature adults—highlighting a developmental shift in the excitation-inhibition balance (Yang et al., 2020). Not all early exposures result in persistent cognitive deficits: some models show mitochondrial dysfunction in the absence of long-term memory impairments, underscoring the importance of exposure pattern and recovery capacity in shaping neurodevelopmental outcomes (Qiu et al., 2024). In adults, context plays a critical role; conditions such as ischemia, inflammation, or metabolic stress can shift the net effect of sevoflurane from protection to injury, consistent with the mechanistic concept that baseline physiological state modulates vulnerability (Qiu et al., 2024; Shen et al., 2013; Sun M. et al., 2021).

Aging: With advancing age, increased inflammatory tone and oxidative stress reduce physiological reserve. In aged rats, SESN2 overexpression attenuates sevoflurane-induced cognitive decline by suppressing NLRP3 inflammasome activation, oxidative damage, and apoptosis—supporting anti-inflammatory and antioxidant strategies as viable perioperative neuroprotective interventions in the elderly (Sun et al., 2024).

Collectively, these stage-specific patterns reinforce the framework introduced earlier: clinical parameters under clinician control—such as anesthetic dose, duration, and timing—intersect with key developmental processes including circuit assembly, myelination, and synaptic pruning, as well as with mechanistic vulnerabilities involving mitochondria, iron-lipid dyshomeostasis, immune-synapse interactions, and pyroptosis. Human brain organoids enable modeling of exposures during fetal-like and neonatal-like developmental windows, allowing assessment of electrophysiological activity, morphological changes, and single-cell molecular states within a human developmental context (Gong et al., 2021; Li et al., 2023; Qian et al., 2019; Trujillo and Muotri, 2018). This provides a human-genetic platform to evaluate age-specific neuroprotective strategies informed by preclinical animal studies (Cornelissen et al., 2018; Lee et al., 2022; Sun M. et al., 2021).

## 3 Overview of brain organoid technology

### 3.1 Fundamental concepts and construction methods of brain organoids

Brain organoids are self-organized, three-dimensional tissues derived from human pluripotent stem cells that recapitulate key milestones of human neurodevelopment—including neuroepithelial formation, progenitor expansion, neuronal differentiation, and early cortical lamination—within a controllable *in vitro* environment (Li et al., 2023; Qian et al., 2019; Trujillo and Muotri, 2018). Compared to monolayer cultures, organoids better preserve native-like cell-cell and cell-matrix interactions and enable multiscale readouts (e.g., morphological dynamics, electrophysiological activity, and single-cell omics) over extended developmental periods, thereby providing enhanced physiological relevance for studies of neural development and toxicological assessment relative to conventional two-dimensional models (Fan et al., 2022; Gong et al., 2021; Zhao and Haddad, 2024) .

Two complementary construction strategies are employed. First, unguided (cerebral organoid) protocols rely on intrinsic patterning to generate multi—regional identities but often exhibit batch-to-batch variability (Lancaster and Knoblich, 2014; Qian et al., 2019; Zhao and Haddad, 2024). Second, guided protocols incorporate temporally controlled morphogen cues (e.g., Dual-SMAD inhibition combined with modulation of the WNT, Sonic hedgehog [SHH], and retinoic acid [RA] signaling axes) to direct cells toward regionally specified fates—such as forebrain, midbrain, hindbrain, or hippocampal lineages—with greater reproducibility (Lancaster and Knoblich, 2014; Qian et al., 2019; Zhao and Haddad, 2024). In practice, many laboratories integrate elements of both approaches to balance cellular diversity with experimental reproducibility (Lancaster and Knoblich, 2014; Qian et al., 2019).

Methodological refinements have significantly enhanced organoid viability and functional maturation. Air-liquid interface (ALI) slice culture, along with related engineering optimizations—such as controlled embryoid body size and shape and sustained basement membrane support—effectively reduces hypoxic core formation, promotes axonal outgrowth, and supports long-term neural network maturation over several months (Giandomenico et al., 2020; Gong et al., 2021). Bioreactors and microfluidic platforms further improve nutrient and oxygen delivery while enhancing experimental control, enabling scalable and prolonged cultures as well as standardized perturbations for robust disease modeling and neurotoxicological studies (Giandomenico et al., 2020; Gong et al., 2021; Nguyen, 2022).

Organoid assembloids expand the experimental toolkit by fusing region-specific organoids or integrating non-neural components (Sabogal-Guaqueta et al., 2024; Velasco et al., 2019; Xiang et al., 2017). For instance, human blood-brain barrier (BBB) assembloids—generated by coupling brain and vascular organoids—recapitulate key structural and functional features of the BBB, including tight junctions, pericyte-astrocyte interactions, and BBB-like transport properties, and can model patient-specific vascular malformations with high transcriptomic and functional fidelity (Dao et al., 2024). These vascularized interfaces, when combined with stretchable mesh microelectronics for chronic recording and stimulation, enable longitudinal electrophysiological phenotyping without requiring tissue flattening or sectioning (Li et al., 2022).

Despite rapid progress, significant challenges remain . Batch-to-batch variability, incomplete cortical lamination and gyrification, limited immune and vascular components, and culture-level maturation plateaus can constrain data interpretation; as a result, community guidelines emphasize protocol standardization, benchmarking against *in vivo* references, and transparent reporting of oxygen and glucose conditions as well as exposure parameters for toxicology applications (Huang et al., 2025; Sandoval et al., 2024; Zhao and Haddad, 2024) . Recent studies demonstrate that organoids can be engineered into reproducible and developmentally faithful systems suitable for *in vitro* neurotoxicity testing—providing practical alternatives when animal-to-human translational gaps or ethical constraints limit traditional approaches (Cao, 2022; Hu et al., 2025; Park and Sun, 2025). Because organoids can be staged and parameterized, they are particularly suited to test sevoflurane exposure paradigms (dose, duration, timing) (Bai, 2024; Lee et al., 2022; Shang et al., 2022).

### 3.2 Existing brain organoid models and their applications

Human brain organoids now encompass a diverse spectrum of model systems that balance cellular diversity, regional specificity, vascular support, and *in vivo* physiological context (Gong et al., 2021; Li et al., 2023). “Unguided” cerebral organoids pioneered the field by demonstrating robust self-organization into cortical-like regions with human-specific progenitor characteristics and enabling patient-specific disease modeling—such as primary microcephaly—that are difficult to recapitulate in rodent models (Lancaster and Knoblich, 2014; Lancaster et al., 2013).

To interrogate defined neural circuits, “guided” protocols generate region-specific organoids—such as cortical or medial ganglionic eminence (MGE)-like tissues—that can be fused into assembloids to investigate long-range cellular interactions, including interneuron migration and integration into cortical networks, under human-specific developmental timing and molecular regulation (Xiang et al., 2017). Large-scale single-cell atlases further demonstrate that, with standardized protocols, organoids can reproducibly generate the major progenitor and neuronal cell types of the human dorsal forebrain, with batch-to-batch variability approaching that of native tissue (Velasco et al., 2019).

Because oxygen availability and immune/vascular niches limit maturation *in vitro*, transplantation of human organoids into the adult mouse brain has been employed to achieve vascularization, prolonged survival, sustained network activity, and functional host–graft synaptic connectivity—providing an *in vivo* complement that expands the physiological scope of organoid research (Mansour et al., 2018). In parallel, microglia-integrated organoids now enable the direct investigation of immune–neural crosstalk in neurodegeneration and brain tumors, addressing questions that are difficult to study in conventional mouse models due to species-specific differences in microglial programs (Sabogal-Guaqueta et al., 2024).

Applications span neurodevelopmental disorders, psychiatric disease mechanisms, and preclinical pharmacology (Dixon and Muotri, 2023; Li et al., 2023). In psychiatric research, organoids provide human-genetic circuit readouts—such as neural oscillations, synaptic physiology, and network maturation—that complement animal models and help bridge behavior-centric diagnoses with underlying cellular mechanisms (Dixon and Muotri, 2023; Jin et al., 2025). In neurodegeneration, patient-derived or genetically engineered organoids have been used to model Alzheimer’s disease (AD)-relevant pathologies and evaluate candidate interventions; recent studies, for example, report reductions in Aβ and p-Tau levels as well as modulation of glial markers following exposure to a glucagon-like peptide-1 receptor agonist (GLP-1RA) in both mouse models and human AD organoid systems—demonstrating how organoids can operationalize drug-response hypotheses within a human cellular context (Zhang et al., 2024).

Across these applications, guidelines emphasize protocol standardization, benchmarking against *in vivo* references, and transparent reporting of media composition, gas conditions, and exposure parameters (Huang et al., 2025; Sandoval et al., 2024). For toxicology workflows, dose, duration, and timing must reflect clinically relevant windows, and organoid platforms are increasingly being optimized for quantitative neurotoxicity testing and drug evaluation (Cao, 2022; Nguyen, 2022; Park and Sun, 2025) . Together, these models constitute a modular toolkit: unguided organoids for broad developmental modeling and patient-specific studies; guided organoids and assembloids for pathway- and circuit-specific investigations; microglia-integrated systems for studying immune–synapse interactions; and *in vivo* engraftment models to enhance maturation and vascular support.

### 3.3 Advantages of brain organoids in toxicological research

In toxicology workflows, brain organoids operationalize mechanism-to-risk translation by coupling human genetics and three-dimensional cytoarchitecture with longitudinal functional readouts, enabling quantitative assessment of sevoflurane exposure across development (Lee et al., 2022; Li et al., 2023; Qian et al., 2019; Trujillo and Muotri, 2018). Unlike monolayer cultures, organoids preserve native-like cell-cell and cell-matrix interactions, support progressive network maturation, and enable multimodal readouts—including imaging, electrophysiology, and single-cell omics—over periods of months (Giandomenico et al., 2020; Gong et al., 2021; Jin et al., 2025; Trujillo and Muotri, 2018). These features are critical for evaluating sevoflurane exposure across clinically relevant windows of synaptogenesis and circuit refinement (Bai, 2024; Lee et al., 2022; Shang et al., 2022). In toxicology workflows, the enhanced physiological fidelity of organoids improves construct validity and aligns outcome measures—such as synaptic integrity, oscillatory activity, and cell-type—specific stress responses—with hypothesized injury pathways previously identified in animal studies: mitochondrial dysfunction-apoptosis, immune-synapse remodeling, iron-lipid dyshomeostasis/ferroptosis, and pyroptosis (Neag et al., 2020; Sun M. et al., 2021).

A second advantage is scalable experimental control. Organoids can be parameterized—by dose, duration, timing, oxygen and glucose levels, and media composition—to model perioperative exposure patterns and to interrogate concentration-response relationships and windows of vulnerability with robust statistical power (Giandomenico et al., 2020; Nguyen, 2022; Qian et al., 2019). Methods chapters and reviews now outline reproducible, region-specific organoid protocols—for forebrain, midbrain, and choroid plexus development—specifically designed for drug testing and toxicological evaluation. These include bioreactors, standardized patterning procedures, and defined maturation media, thereby enabling harmonized protocols and cross-laboratory comparability (Giandomenico et al., 2020; Li et al., 2023; Nguyen, 2022; Park and Sun, 2025). Complementary guidance from dedicated toxicology overviews emphasizes assay standardization, batch-to-batch quality metrics, and readiness for high-throughput screening, positioning neural organoids as a scalable screening-tier platform rather than merely a bespoke research tool (Cao, 2022; Fan et al., 2022; Park and Sun, 2025).

Third, organoids have demonstrated face validity across a range of neurotoxicants, supporting their utility as human-relevant sentinels prior to advancing to animal or clinical studies (Cao, 2022; Fan et al., 2022; Hu et al., 2025; Park and Sun, 2025). For example, cerebral organoids exposed to silver nanoparticles show dose-dependent developmental disruptions—including impaired cilia assembly, cytoskeletal disorganization, and imbalances in proliferation and apoptosis—that are difficult to detect in two-dimensional cultures, illustrating the ability of organoids to capture tissue-level vulnerabilities with cell-type specificity (Huang et al., 2022). Converging reviews summarize these applications and highlight emerging advancements—such as barrier-containing or vascularized constructs, assembloids, and microphysiological systems—that broaden the spectrum of toxicological endpoints (e.g., transport and permeability, immune-neural crosstalk) and enhance translational relevance (Cao, 2022; Li et al., 2023; Park and Sun, 2025).

Finally, organoids facilitate the operationalization of mitigation testing within a human genetic context (Bai, 2024; Li et al., 2023). By preserving developmental tempo and cellular diversity, organoids enable stress-testing of mechanism-based interventions derived from sevoflurane studies—such as iron/ferroptosis modulators, anti-inflammatory strategies targeting complement/TREM2 or sialidases, and approaches aimed at stabilizing mitochondrial dynamics or restoring ER-mitochondrial coupling and mitophagy homeostasis (Gao and Huang, 2024; Kang et al., 2023; Sun M. et al., 2021; Wang et al., 2024). When integrated with standardized exposure paradigms and shared analytical frameworks, organoids can transform the mechanistic hypotheses outlined in Sections “1 Introduction” and “2 Effects of sevoflurane on the nervous system at different developmental stages” into quantitative measures of risk and rescue efficacy that are amenable to benchmarking and replication . Collectively, these attributes establish brain organoids as a human-relevant, scalable, and mechanistically informative platform for assessing sevoflurane-induced neurotoxicity within the framework of evidence-based perioperative safety research.

## 4 Effects of sevoflurane on brain organoids

### 4.1 Morphological and histological alterations in brain organoids following sevoflurane exposure

**FIGURE 1:**
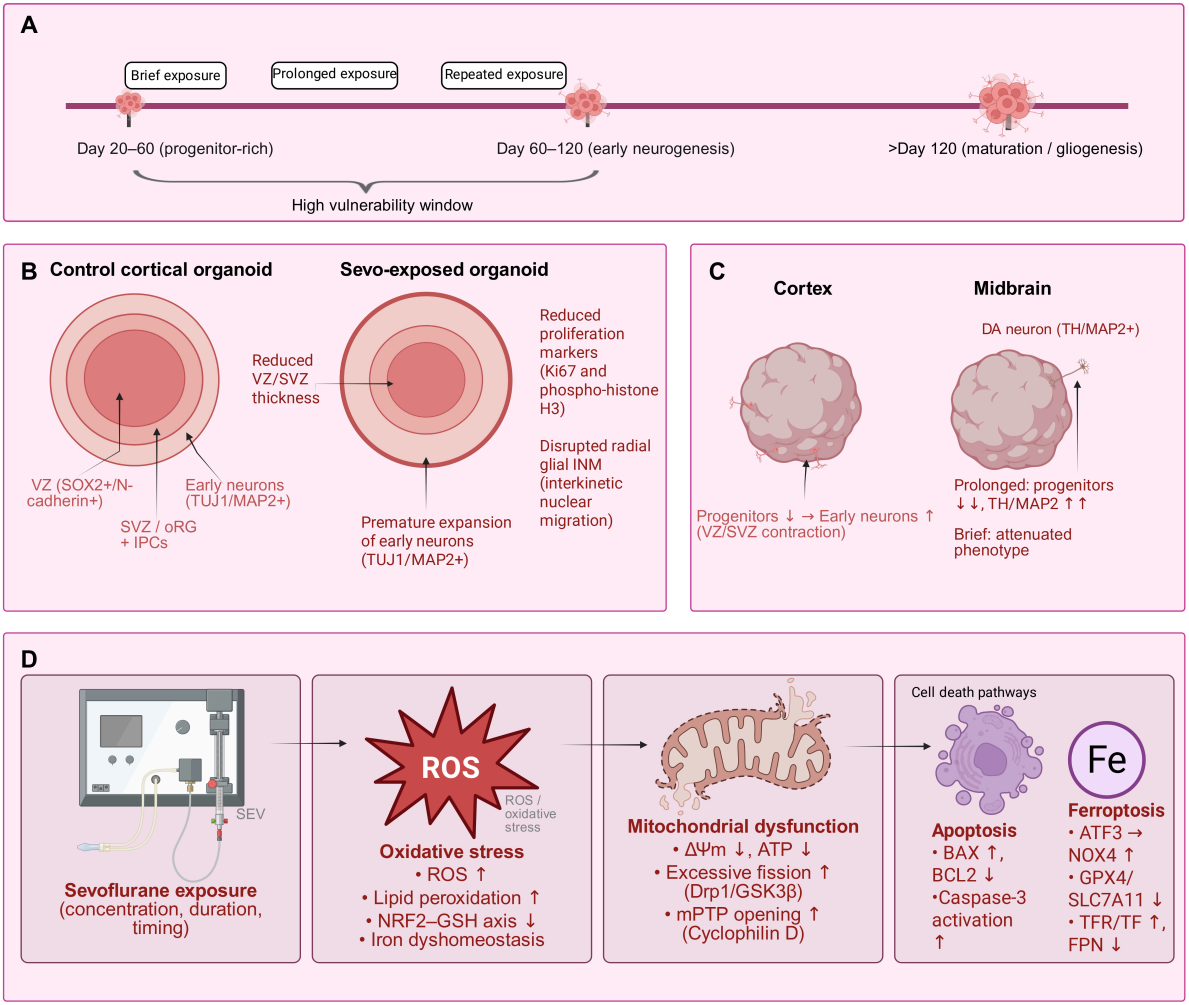
Organoid-based framework linking dose–duration–timing of sevoflurane exposure to morphological and mechanistic endpoints. **(A)** Schematic dose–duration–timing framework of sevoflurane exposure mapped onto developmental windows in human brain organoids, highlighting a high vulnerability window spanning progenitor-rich and early neurogenic stages (day 20–120) and experimentally programmable brief, prolonged, and repeated exposure paradigms. **(B)** Cross-sections of cortical organoids illustrating progenitor-zone instability after sevoflurane exposure: thinning of VZ/SVZ progenitor zones (SOX2^+^/N-cadherin^+^), reduced proliferation (Ki67 and phospho-histone H3), disrupted radial glial interkinetic nuclear migration (INM), and premature expansion of the TUJ1/MAP2^+^ neuronal layer. **(C)** Region- and lineage-specific consequences in cortical versus midbrain organoids. In cortical organoids, sevoflurane shifts the balance from progenitor expansion toward early neurons with VZ/SVZ contraction. In midbrain organoids, prolonged exposure depletes progenitors and accelerates dopaminergic maturation (TH/MAP2^+^), whereas brief exposure produces attenuated phenotypes. **(D)** Oxidative stress–mitochondrial dysfunction–cell death cascade. Sevoflurane exposure (with controlled concentration, duration and timing) increases ROS and lipid peroxidation, impairs NRF2–GSH signaling and iron homeostasis, leading to mitochondrial dysfunction (loss of ΔΨm, reduced ATP, excessive GSK3β/Drp1-mediated fission, and Cyclophilin-D–dependent mPTP opening) and convergence onto apoptosis (BAX↑, BCL2↓, caspase-3 activation) and ferroptosis (ATF3→NOX4, GPX4/SLC7A11↓, TFR/TF↑, FPN↓).

Organoid-based studies demonstrate that sevoflurane does not induce a uniform cytopathic effect; instead, it selectively disrupts critical developmental checkpoints that govern the spatiotemporal organization of tissue architecture (Lee et al., 2022; see Figure 1A). In cerebral organoids exposed to sevoflurane during progenitor-rich developmental windows, ventricular-like zones (VZs), subventricular-like zones (SVZ), outer radial glia (oRG), and intermediate progenitor cells (IPCs) exhibit structural instability, characterized by impaired interkinetic nuclear migration (INM) in radial glial cells, reduced mitotic activity at the apical surface, and thinning or focal disorganization of the SOX2+/N-cadherin+ progenitor layer (Gong et al., 2021; Lee et al., 2022; see Figure 1B). Concomitantly, early neuronal markers (TUJ1/MAP2) are prematurely expressed, coinciding with a decline in proliferative indicators such as Ki67 and phospho-histone H3 (PH3), suggesting an accelerated transition from progenitor expansion to neuronal differentiation (Lee et al., 2022). Although rudimentary cortical layering may partially recover during prolonged culture, the transient disruption of cellular composition and laminar organization delineates a vulnerable developmental period during which neural circuit formation may be durably retuned (Giandomenico et al., 2020; Li et al., 2023).

Mechanistic insights are anchored in studies focused on interkinetic nuclear migration (INM) conducted in both fetal cortex and human embryonic stem cell (ESC)-derived organoids: sevoflurane transiently disrupts radial glial INM in a Notch-dependent manner, as activation of the Jag1/NICD pathway mitigates this impairment (Jiang et al., 2021). The consistent observation of this phenotype across *in utero* and organoid models supports the interpretation that INM represents a developmentally significant vulnerability node relevant to human neurodevelopment, aligning with the “dose-duration-timing” framework emphasized in Sections “1 Introduction” and “2 Effects of sevoflurane on the nervous system at different developmental stages” (Jiang et al., 2021; Lee et al., 2022). A similar pattern of premature differentiation is observed in region-specific midbrain organoids, where prolonged exposure reduces the progenitor pool while accelerating dopaminergic lineage commitment, evidenced by earlier and enhanced expression of MAP2/TH markers (Shang et al., 2022). In contrast, shorter exposure durations attenuate these effects, underscoring a duration-dependent response that parallels clinical perioperative practices (Shang et al., 2022; see Figure 1C).

At the tissue-patterning level, cortical-like organoids consistently exhibit contraction of ventricular-like and subventricular-like zones (VZ/SVZ), accompanied by a transient expansion of early neuronal layers, followed by a partial restoration of inside-out cortical lamination (Gong et al., 2021; Lee et al., 2022; see Figure 1B). When these organoid phenotypes are integrated with system-level mechanisms outlined in Section “2 Effects of sevoflurane on the nervous system at different developmental stages,” a convergent pathological framework emerges: instability of the progenitor zones coincides temporally and spatially with accelerated neuronal differentiation and is associated with multiple stress-related pathways, including dysregulation of mitochondrial dynamics and apoptosis, iron-lipid imbalance with features of ferroptosis, and immune-synapse remodeling (with *in vivo* support in rodent models) (Kang et al., 2023; Sun M. et al., 2021; Wang et al., 2024; see Figure 1D). Histological evidence supports this association, revealing focal increases in apoptotic cells within progenitor zones, microdomain-specific depletion of outer radial glia and intermediate progenitor cells (oRG/IPCs), and the premature appearance of spine-bearing neurons within domains normally progenitor-dominant at matched developmental ages (Deng et al., 2024; Lee et al., 2022; Sun M. et al., 2021).

Importantly, the trajectory of these alterations is highly dependent on experimental context (Li et al., 2023). Organoids cultured under air-liquid interface conditions, in bioreactors, or with enhanced oxygen and glucose delivery exhibit improved long-term viability and a greater propensity for partial architectural recovery, indicating that certain changes may represent reversible developmental pauses rather than fixed structural loss (Giandomenico et al., 2020; Gong et al., 2021; Hu et al., 2025). Nevertheless, even when laminar organization re-emerges, earlier perturbations—such as altered progenitor kinetics or imbalanced neuron-to-glia ratios—may induce hysteresis in neural circuit assembly, which remains undetectable in short-term assessments . These context-dependent trajectories align with observed clinical heterogeneity and underscores the necessity of mapping dose, duration, and timing onto key developmental milestones—including synaptogenesis, phases of INM, and the onset of gliogenesis—when evaluating neurodevelopmental risk (Hu et al., 2025; Zhao and Haddad, 2024; see Figure 1A).

Region and lineage-specific consequences further enhance translational relevance (Lee et al., 2022; Shang et al., 2022). In midbrain organoids, sevoflurane promotes premature dopaminergic maturation while reducing the proliferative compartment; in cortical organoids, it narrows the apical progenitor zone and accelerates neuronal marker expression (Jiang et al., 2021; Shang et al., 2022; see Figure 1C). Across model systems, systematic modulation of dose, duration, and timing influences effect magnitude, providing a controlled experimental framework to recapitulate clinically relevant scenarios—such as brief induction versus prolonged maintenance anesthesia, or single versus repeated anesthetic exposures—and to evaluate mitigation strategies outlined in Section “2 Effects of sevoflurane on the nervous system at different developmental stages,” including iron/ferroptosis modulators, complement/TREM2-targeted anti-inflammatory interventions, and agents preserving mitochondrial dynamics, with histological endpoints serving as tightly coupled outcome measures (Kang et al., 2023; Lee et al., 2022; Shang et al., 2022; Sun M. et al., 2021).

Collectively, the organoid literature supports a unifying morphological paradigm—progenitor-zone instability coupled with region-specific premature differentiation, with partial reversibility achievable under optimized culture conditions (Lee et al., 2022; Li et al., 2023; see Figures 1B, C). This paradigm extends the multi-node injury framework described in Section “2 Effects of sevoflurane on the nervous system at different developmental stages” into a human genetic, three-dimensional context, transforming mechanistic hypotheses into quantifiable histological endpoints such as VZ/SVZ thickness, interkinetic nuclear migration INM coherence, progenitor cell indices, and the timing of layer-specific markers (Lee et al., 2022; Li et al., 2023; Qian et al., 2019; Sun M. et al., 2021). These measurable outcomes can be systematically benchmarked across dose–duration–timing matrices to inform evidence-based perioperative safety assessments (Lee et al., 2022; Li et al., 2023; Qian et al., 2019; Shang et al., 2022; see Figures 1A–D).

### 4.2 Impact of sevoflurane on neural development and functional maturation in brain organoids

Leveraging the human genetic background and protracted developmental tempo of brain organoids, recent studies converge on a stage-dependent paradigm: sevoflurane disrupts early neurogenic checkpoints that establish cortical and midbrain architecture, and these cellular perturbations presage subsequent network-level alterations (Lee et al., 2022; Qian et al., 2019; Shang et al., 2022; Trujillo and Muotri, 2018; see Figures 1A–C). In cortical organoids exposed during progenitor-enriched developmental windows, ventricular-like and subventricular-like zones contract and exhibit disrupted interkinetic nuclear migration (INM) in radial glia; apical mitoses are reduced, progenitor layers become disorganized, and early neuronal markers (e.g., TUJ1, MAP2) emerge prematurely—collectively supporting an accelerated shift from proliferative expansion to neuronal differentiation (Giandomenico et al., 2020; Gong et al., 2021; Lee et al., 2022). Mechanistically, transient INM defects are linked to Notch pathway dependence: activation of Jagged1/Notch intracellular domain signaling has been shown to restore migration dynamics, underscoring INM as a human-relevant vulnerability node consistent with the previously introduced “dose–duration–timing” exposure framework (Jiang et al., 2021; Lee et al., 2022).

A complementary pattern is observed in region-specified midbrain organoids (Shang et al., 2022). Prolonged exposure reduces the progenitor pool and accelerates dopaminergic lineage commitment, accompanied by earlier and stronger expression of MAP2 and tyrosine hydroxylase (TH); in contrast, shorter exposures attenuate these changes, revealing a duration-dependent phenotypic response that is conceptually parallel to clinical distinctions between brief induction and prolonged maintenance anesthesia (model-based extrapolation rather than a direct clinical inference) (Patel and Goa, 1996; Shang et al., 2022; Warner et al., 2018; Xiao et al., 2022; see Figure 1C). Together with cortical findings, these results support a unifying morphological paradigm: progenitor-zone instability coupled with region-specific premature differentiation, rather than a uniform cytopathic effect (Gong et al., 2021; Lee et al., 2022; Shang et al., 2022; see Figures 1B, C). This paradigm aligns with the upstream injury mechanisms outlined in Section “2 Effects of sevoflurane on the nervous system at different developmental stages”—mitochondrial stress and apoptosis, immune–synapse remodeling, and iron-lipid dyshomeostasis leading to ferroptosis (Kang et al., 2023; Sun M. et al., 2021; Wang et al., 2024)—and provides quantifiable histological readouts, including VZ/SVZ thickness, INM coherence, and progenitor indices, which can be systematically benchmarked across controlled exposure matrices (Gong et al., 2021; Lee et al., 2022; Li et al., 2023).

Functionally, organoids provide a sufficiently extended temporal window to link acute cellular alterations with later network-level behaviors (Giandomenico et al., 2020; Jin et al., 2025; Trujillo and Muotri, 2018). In long-term cultures spanning several months, multimodal assays—including live imaging, microelectrode arrays (MEAs), and single-cell omics—offer a standardized framework to detect changes in neural rhythm and synchrony, thereby enabling studies that connect premature neuronal activity to altered oscillatory dynamics and synaptic organization (Dixon and Muotri, 2023; Giandomenico et al., 2020; Jin et al., 2025; Velasco et al., 2019). While not every sevoflurane–organoid study has yet reported a full electrophysiological profile, these methods are now established and applicable, and the emerging functional signatures are consistent with the developmental framework outlined in Sections “1 Introduction” and “2 Effects of sevoflurane on the nervous system at different developmental stages”: when differentiation proceeds at the expense of progenitor renewal, neural circuits assemble on an imbalanced cellular substrate, increasing the likelihood that early, partially reversible histological deviations become permanently embedded as persistent network “scars” (Giandomenico et al., 2020; Lee et al., 2022; Qian et al., 2019; Trujillo and Muotri, 2018).

Crucially, organoids also enable the operationalization of mitigation testing within a human genetic context (Lee et al., 2022; Li et al., 2023; Qian et al., 2019). Because exposure parameters are programmable—including concentration, duration, and oxygen/glucose levels—researchers can apply mechanistic interventions such as iron/ferroptosis modulators, complement/TREM2-targeted anti-inflammatory strategies, or approaches that stabilize mitochondrial dynamics and ER–mitochondria coupling (Kang et al., 2023; Liu et al., 2022; Sun M. et al., 2021; Wang et al., 2024). Functional rescue can then be assessed through coordinated morphological and electrophysiological endpoints in organoids, including changes in progenitor dynamics, synaptic organization, and network activity (Giandomenico et al., 2020; Jin et al., 2025; Li et al., 2023; see Figure 1D). This explicitly closes the loop with Sections “1 Introduction” and “2 Effects of sevoflurane on the nervous system at different developmental stages,” transforming the mechanistic framework—apoptosis, immune–synapse dysregulation, ferroptosis, and pyroptosis—into quantitative risk and rescue profiles within a platform that bridges preclinical discovery and perioperative decision-making (Dai et al., 2021; Lee et al., 2022; Sun M. et al., 2021).

### 4.3 Potential toxic mechanisms: oxidative stress, mitochondrial dysfunction, and apoptosis

Across human brain organoids and complementary animal models, sevoflurane’s developmental neurotoxicity converges on three interconnected mechanisms—oxidative stress, mitochondrial dysfunction, and programmed cell death—modulated by dose, duration, and developmental timing (Miao et al., 2024; Sun M. et al., 2021; Trujillo and Muotri, 2018; see Figure 1D). While Sections “3 Overview of brain organoid technology” and “4.2 Impact of sevoflurane on neural development and functional maturation in brain organoids” outline why organoids enable parameterized exposure and multimodal readouts, here we focus on the molecular cascade that links those experimental levers to developmental risk. Brain organoids are particularly valuable in this context: their human genetic background and extended maturation period enable the identification of how early biochemical disturbances can propagate into altered neuronal lineage specification and disrupted network development (Qian et al., 2019; Trujillo and Muotri, 2018).

#### 4.3.1 Oxidative stress

In organoid models, sevoflurane has been repeatedly observed to shift the redox balance toward reactive oxygen species (ROS) accumulation, lipid peroxidation, and depletion of antioxidants—a pattern most prominent in proliferative zones and nascent neuronal layers (Cao, 2022; Oyefeso et al., 2021). These redox alterations are associated with the downregulation of cytoprotective pathways, such as the NRF2–GSH axis, and disruptions in iron homeostasis, both of which increase membrane susceptibility to peroxidation and elevate vulnerability during human-specific periods of neurogenesis (Lee et al., 2022; Miao et al., 2024). Conceptually, this positions oxidative stress not as an isolated insult but as an upstream driver that promotes mitochondrial instability and activates cell death programs in a developmentally dependent manner (Liu et al., 2022; Qiu et al., 2024; Sun M. et al., 2021).

#### 4.3.2 Mitochondrial dysfunction

Mitochondria serve as a central hub integrating redox stress and cellular fate instability (Liu et al., 2022; Qiu et al., 2024; Sun M. et al., 2021). In developing systems, sevoflurane impairs mitochondrial membrane potential, reduces ATP synthesis, promotes excessive fission, and lowers the threshold for mitochondrial permeability transition (Liu et al., 2022; Qiu et al., 2024; Sun M. et al., 2021). Data from neural progenitor cell (NPC) migration assays demonstrate that Cyclophilin-D-dependent mitochondrial permeability contributes to compromised progenitor motility—a process with direct consequences for cortical lamination and the timing of neuronal layer assembly (Lu et al., 2023). Organoid studies corroborate these observations: disruption of progenitor bioenergetics perturbs ventricular and subventricular zone dynamics as well as interkinetic nuclear migration, ultimately leading to premature neuronal differentiation and altered network maturation (Lee et al., 2022; Shang et al., 2022). Mechanistically, the mitochondria-centered signaling axis implicated in neonatal rodent cognitive outcomes—including GSK3β/Drp1 activation and ER-mitochondria coupling provides a plausible explanation for how brief and prolonged exposures yield qualitatively distinct neurodevelopmental effects (Liu et al., 2022; Qiu et al., 2024; Zhang et al., 2022).

#### 4.3.3 Apoptosis and ferroptosis

Programmed cell death arises downstream of these stressors, not as a singular pathway but through multiple coordinated mechanisms (Kang et al., 2023; Sun M. et al., 2021; Zeng et al., 2024). In both organoid and *in vivo* models, sevoflurane activates apoptosis—characterized by BAX upregulation, BCL2 downregulation, and caspase-3 activation—concurrently with ferroptosis, an iron-dependent form of lipid peroxidation that is particularly prevalent in immature neural tissues rich in polyunsaturated fatty acids (PUFAs) and deficient in antioxidant defenses (Kang et al., 2023; Sun M. et al., 2021; Zeng et al., 2024). Recent studies have identified an ER-stress–induced ATF3→NOX4 axis that amplifies H2O2 production, suppresses GPX4 and SLC7A11 expression, and promotes iron overload signatures—including increased transferrin receptor (TFR) and transferrin (TF), and decreased ferroportin (FPN)—thereby predisposing developing neurons to ferroptosis (Kang et al., 2023; Zeng et al., 2024). These findings integrate redox imbalance, endoplasmic reticulum stress, and dysregulated iron homeostasis into a coherent ferroptotic module that coexists with apoptotic pathways and may become predominant under specific exposure conditions (Kang et al., 2023; Sun M. et al., 2021).

#### 4.3.4 Why organoids matter

By systematically controlling exposure parameters—concentration, duration, and timing—and measuring orthogonal endpoints such as redox indices, mitochondrial function, apoptosis and ferroptosis markers, progenitor dynamics, and network electrophysiology, organoids transform mechanistic insights into quantifiable developmental risk (Fan et al., 2022; Qian et al., 2019; Trujillo and Muotri, 2018). They further enable intervention testing—for instance, with antioxidants, ferroptosis modulators (including iron chelators or agents that support GPX4/SLC7A11), or mitochondrial stabilizers—allowing injury-related phenotypes to be functionally rescued and benchmarked within a human developmental framework (Fan et al., 2022; Huang et al., 2022; Lu et al., 2023). Collectively, these capabilities bridge the biological mechanisms outlined in Sections “1 Introduction” and “2 Effects of sevoflurane on the nervous system at different developmental stages” with a translational pipeline for improving perioperative neurodevelopmental safety .

## 5 Brain organoid platforms as a framework for risk quantification and translational assessment

### 5.1 Experimental standardization and exposure parameterization

A central challenge in evaluating sevoflurane-associated neurotoxicity lies in the non-binary nature of risk, which is dynamically shaped by multiple exposure parameters—including concentration, duration, timing relative to key neurodevelopmental milestones, and physiological context (such as oxygenation and metabolic support)—all of which exhibit substantial variability across experimental studies and clinical practice (Miao et al., 2024; Patel and Goa, 1996; Sun M. et al., 2021). Brain organoids provide a uniquely programmable platform in which these variables can be systematically defined, precisely controlled, and directly compared (Fan et al., 2022; Qian et al., 2019; Trujillo and Muotri, 2018). By staging organoids to align with specific developmental windows—mimicking fetal, neonatal, or early postnatal stages—researchers can model clinically representative exposure scenarios, such as brief induction-like versus prolonged maintenance-like exposures, or single versus repeated anesthetic episodes, and robustly correlate these regimens with morphological, molecular, and functional outcomes (Lee et al., 2022; Qian et al., 2019; Shang et al., 2022; Trujillo and Muotri, 2018). This capability overcomes a persistent limitation of rodent and in utero models, where manipulation of exposure timing and duration is constrained by maternal physiology, pharmacokinetic variability, and ethical considerations regarding repetitive interventions. In contrast, organoids enable high-throughput, parallel assessment of comprehensive “dose-duration-timing” matrices under tightly controlled conditions, thereby facilitating more rigorous and reproducible risk evaluation (Fan et al., 2022; Qian et al., 2019; Sandoval et al., 2024).

From a methodological standpoint, this parameterization hinges on rigorous standardization (Fan et al., 2022; Sandoval et al., 2024). Current community guidance and protocols emphasize clear reporting of exposure concentrations, the timing of exposure relative to organoid developmental age, media composition, oxygen and glucose levels, and batch identity—each of which can significantly influence cellular vulnerability and recovery trajectories (Hu et al., 2025; Sandoval et al., 2024; Zhao and Haddad, 2024). These standardization requirements mirror clinical insights already established in perioperative medicine: anesthetic risk cannot be determined by drug identity alone, but arises from the interplay between drug administration and the recipient’s specific developmental and physiological context (Patel and Goa, 1996; Sun M. et al., 2021). In this regard, brain organoids serve not only as mechanistic models of neurodevelopmental toxicity but also as quantitative calibration tools. They enable researchers to determine whether a given exposure regimen remains within a biologically tolerable threshold for a particular developmental stage or instead crosses into pathological regimes associated with radial glial proliferative collapse, premature neuronal differentiation, or persistent network dysfunction (Fan et al., 2022; Hu et al., 2025; Lee et al., 2022).

### 5.2 Multimodal readouts and quantitative neurodevelopmental risk profiling

A second key advantage of organoid platforms is their capacity to support multiscale, multimodal assessment of developmental outcomes over time (Giandomenico et al., 2020; Gong et al., 2021; Lancaster and Knoblich, 2014). As detailed in Section “4 Effects of sevoflurane on brain organoids,” sevoflurane exposure in cortical-like and midbrain-like organoids consistently induces a characteristic neurodevelopmental phenotype: contraction or structural disorganization of progenitor zones (including ventricular-like and subventricular-like regions), impaired interkinetic nuclear migration (INM) in radial glia, reduced apical mitotic activity, and precocious expression of neuronal markers (e.g., TUJ1, MAP2, TH)—collectively indicating premature lineage commitment at the expense of progenitor self-renewal (Hu et al., 2025; Lee et al., 2022; Shang et al., 2022). Because these phenotypic alterations are quantifiable—such as ventricular zone thickness, Ki67/PH3-based proliferation indices, INM coherence, and the timing of dopaminergic or cortical neuron marker emergence—they can be integrated into semi-quantitative vulnerability scores that capture the degree to which a given exposure disrupts normal developmental tempo (Fan et al., 2022; Li et al., 2023; Sandoval et al., 2024).

These structural and lineage-level endpoints can be integrated with molecular stress signatures to provide a more comprehensive assessment of neurodevelopmental toxicity (Fan et al., 2022; Li et al., 2023; Neag et al., 2020; Sun M. et al., 2021). Organoids enable concurrent profiling of oxidative stress and ferroptosis markers (including ROS accumulation, lipid peroxidation, dysregulated iron metabolism, and downregulation of GPX4/SLC7A11), mitochondrial stress and imbalances in fission/mitophagy (such as GSK3β/Drp1 activation, loss of mitochondrial membrane potential, and defects in ER-mitochondria coupling), and inflammation-associated pathways (including NF-κB activation, inflammasome assembly, complement-mediated tagging and microglia-like phagocytosis of synapses) (Kang et al., 2023; Liu et al., 2022; Sun M. et al., 2021; Xu et al., 2023). By integrating multiple biological axes—progenitor cell stability, differentiation timing, bioenergetic homeostasis, ferroptotic vulnerability, and synaptic/immune remodeling—organoids generate a multidimensional injury profile that extends far beyond a binary “cell death yes/no” readout (Fan et al., 2022; Li et al., 2023). This integrative approach is critical because, as detailed in Sections “2 Effects of sevoflurane on the nervous system at different developmental stages” and “4 Effects of sevoflurane on brain organoids,” sevoflurane-induced developmental neurotoxicity encompasses not only acute apoptosis but also long-term disruptions such as aberrant neural circuit assembly, maladaptive synaptic pruning, and metabolic reprogramming of progenitor pools—alterations that may only become functionally evident weeks after exposure (Briner et al., 2010; Sun M. et al., 2021; Wang et al., 2024; Zhao et al., 2022).

Finally, organoids mature sufficiently to enable functional readouts—such as spontaneous neural activity, oscillatory coupling, synchrony across neuronal clusters, and electrophysiological responsiveness measured via microelectrode arrays or integrated bioelectronic meshes (Giandomenico et al., 2020; Jin et al., 2025; Li et al., 2022)—thereby linking early architectural and molecular disruptions to later network-level functional outcomes (Dixon and Muotri, 2023; Qian et al., 2019; Trujillo and Muotri, 2018). This temporal continuity allows researchers to differentiate between transient, self-correcting perturbations (e.g., a reversible delay in interkinetic nuclear migration) and persistent “developmental scars,” such as sustained network desynchronization or disrupted excitatory-inhibitory (E-I) balance (Giandomenico et al., 2020; Jin et al., 2025; Mansour et al., 2018; Velasco et al., 2019). Practically speaking, this capability enables organoids to generate a quantitative risk profile for a given exposure paradigm: not merely whether a change occurred, but whether that change is likely to endure and impair circuit-level function (Dixon and Muotri, 2023; Fan et al., 2022; Li et al., 2023; Trujillo and Muotri, 2018). This distinction holds significant clinical relevance (Cornelissen et al., 2018; Patel and Goa, 1996; Sun M. et al., 2021; Yang et al., 2020).

### 5.3 Toward translational integration: from developmental windows to perioperative decision-making

The convergence of controlled exposure paradigms and multidimensional readouts establishes brain organoids as a translational interface between mechanistic toxicology and perioperative medicine (Fan et al., 2022; Li et al., 2023; Qian et al., 2019; Trujillo and Muotri, 2018). In clinical practice, anesthesiologists already individualize care based on developmental stage, procedural urgency, comorbid conditions, and expected anesthetic duration—particularly in neonates, infants, and fetuses, whose brains are actively undergoing critical neurodevelopmental processes such as progenitor expansion, cortical lamination, axonal targeting, synaptic pruning, and network synchronization (Cornelissen et al., 2018; Warner et al., 2018; Xiao et al., 2022). What has been lacking is a human-relevant preclinical model capable of prospectively predicting how a given exposure regimen impacts these dynamic developmental programs (Bai, 2024; Cao, 2022; Qian et al., 2019; Trujillo and Muotri, 2018). Brain organoids represent a promising step toward addressing this unmet need (Li et al., 2023; Qian et al., 2019; Trujillo and Muotri, 2018).

First, because organoids can be precisely staged, they enable the alignment of exposure timing with key neurodevelopmental milestones (Giandomenico et al., 2020; Lancaster and Knoblich, 2014; Velasco et al., 2019). For instance, exposing cortical-like organoids during the peak period of radial glial proliferation and ventricular zone expansion recapitulates a fetal or early neonatal developmental window, whereas exposing more mature midbrain organoids during robust dopaminergic differentiation mirrors later neonatal or infant stages (Lancaster and Knoblich, 2014; Lee et al., 2022; Shang et al., 2022; Velasco et al., 2019). Second, because the same platform can be subjected to multiple exposure regimens, it allows for direct comparison of clinically distinct scenarios—such as brief single anesthesia, prolonged maintenance anesthesia, and repeated exposures across several days—and reveals which regimen most severely compromises progenitor integrity, accelerates premature neuronal differentiation, disrupts mitochondrial homeostasis, or induces inflammatory and synaptic pruning phenotypes (Lee et al., 2022; Shang et al., 2022; Sun M. et al., 2021; Wang et al., 2024; Zhao et al., 2022). This ability to map anesthetic exposure patterns onto stage-specific biological vulnerabilities is, in principle, applicable to perioperative risk assessment and future clinical decision-making, provided that organoid-based endpoints are sufficiently standardized and validated in prospective translational studies (Sandoval et al., 2024; Xiao et al., 2022).

Third, because organoids can incorporate vascular-like interfaces and barrier properties, astroglia, and microglia-like populations, and can be profiled at single-cell resolution, they offer a feasible approach to stratifying developmental vulnerability across distinct cell types and neuroanatomical compartments, rather than treating the pediatric brain as a uniform target (Buonfiglioli et al., 2025; Dao et al., 2024; Hu et al., 2025; Sabogal-Guaqueta et al., 2024). This capability lays the foundation for future precision medicine strategies: identifying high-risk developmental periods—such as peak radial glial proliferation—during which clinicians might prioritize minimizing cumulative anesthetic exposure; defining lower-risk windows in which brief exposures are unlikely to result in persistent network-level deficits; and ultimately, screening candidate perioperative co-interventions—such as compounds that stabilize mitochondrial dynamics, modulate ferroptosis susceptibility, or suppress complement-mediated synaptic pruning—directly within human-derived systems prior to clinical translation (Buonfiglioli et al., 2025; Li et al., 2023; Sandoval et al., 2024; Trujillo and Muotri, 2018).

To date, sevoflurane–organoid studies have remained largely mechanistic and preclinical, with no existing studies directly linking organoid-derived injury signatures to long-term neurodevelopmental outcomes or circulating biomarkers in pediatric populations exposed to anesthesia (Lee et al., 2022; Sandoval et al., 2024; Shang et al., 2022). Therefore, the translational framework outlined here should be regarded as a conceptual and methodological foundation rather than an established clinical instrument.

In this way, brain organoids transform the conceptual challenge introduced in Section “1 Introduction”—how to reconcile sevoflurane’s essential clinical utility with its potential developmental neurotoxicity—into an experimentally tractable workflow (Bai, 2024; Fan et al., 2022; Li et al., 2023; Trujillo and Muotri, 2018). Sections “2 Effects of sevoflurane on the nervous system at different developmental stages” and “4 Effects of sevoflurane on brain organoids” established that sevoflurane-induced neurotoxicity is multifactorial, involving mitochondrial stress, ferroptosis, immune-synaptic remodeling, and premature neuronal differentiation, and is developmentally regulated, with outcomes critically dependent on dose, duration, and timing (Kang et al., 2023; Lee et al., 2022; Sun M. et al., 2021; Wang et al., 2024). Section “5 Brain organoid platforms as a framework for risk quantification and translational assessment” outlines a systematic framework for quantifying, staging, and comparing these adverse effects in a human-relevant model, generating exposure-response relationships and stage-specific vulnerability profiles that could eventually inform perioperative risk assessment in a more quantitative and developmentally specific manner once anchored to clinical outcome and biomarker data (Cao, 2022; Fan et al., 2022; Li et al., 2023; Sandoval et al., 2024). This sets up Section “6 Future directions and perspectives” to address platform optimization, remaining methodological limitations, and the long-term neurodevelopmental implications of pediatric exposure (Li et al., 2023; Sandoval et al., 2024; Zhao and Haddad, 2024).

## 6 Future directions and perspectives

### 6.1 Optimization and advancement of brain organoid technology

A credible organoid platform for assessing anesthetic neurotoxicity must first address the “two Rs”—rigor and reproducibility (Sandoval et al., 2024). Substantial inter-laboratory variability in pluripotent stem cell lines, passage history, media composition, oxygenation conditions, and batch identity continues to introduce significant heterogeneity in progenitor cell populations and maturation trajectories, thereby confounding the detection of genuine drug-induced effects (Li et al., 2023; Sandoval et al., 2024; Zhao and Haddad, 2024). To mitigate these challenges, the next phase of research should require a standardized minimum reporting framework encompassing cell line provenance and karyotypic stability, use of independent differentiation batches, precise developmental staging at the time of exposure, monitoring of dissolved oxygen and glucose levels, and pre-specified statistical power and replication strategies (Li et al., 2023; Sandoval et al., 2024; Zhao and Haddad, 2024). Such standardization would enable reliable differentiation between true sevoflurane-associated phenotypes—such as ventricular zone (VZ) or subventricular zone (SVZ) thinning and premature neuronal marker expression—and artifacts arising from culture variability (Lee et al., 2022; Shang et al., 2022). From a methodological standpoint, this further supports the implementation of stage-locking protocols to synchronize organoid development to defined fetal or early infant-like developmental windows prior to exposure, as well as the establishment of shared benchmark datasets analyzed through blinded, harmonized pipelines to ensure cross-site comparability of effect sizes (Giandomenico et al., 2020; Li et al., 2023; Sandoval et al., 2024).

Engineering the microenvironment constitutes the second foundational pillar (Gong et al., 2021). Persistent challenges such as necrotic core formation and diffusion limitations continue to restrict organoid maturation and skew experimental readouts toward early neurogenic events (Gong et al., 2021; Lancaster and Knoblich, 2014). Advanced vascularization and perfusion strategies—including endothelial co-culture, microfluidic systems with continuous perfusion, and blood-brain barrier (BBB)-like assembloids—enhance oxygen and glucose delivery while improving metabolic waste clearance (Dao et al., 2024; Giandomenico et al., 2020). These improvements extend the viable tissue thickness and permit the assessment of later neurodevelopmental milestones under sevoflurane exposure, such as gliogenesis and synaptogenesis (Giandomenico et al., 2020; Gong et al., 2021). In contrast to “chemical acceleration” approaches—such as broad Notch pathway inhibition—microenvironment-first engineering preserves endogenous developmental programs while reducing the time required to reach assayable stages, thereby achieving a more favorable balance between biological fidelity and experimental throughput in neurotoxicity screening (Cao, 2022; Giandomenico et al., 2020; Gong et al., 2021).

Immune competence represents a third critical frontier in organoid modeling. Given that microglia critically regulate synapse formation and complement-mediated synaptic pruning—key pathways implicated in anesthetic-induced neurotoxicity—future models should routinely integrate iPSC-derived microglia or hematopoietic progenitor cell-based seeding protocols to recapitulate neuroimmune interactions (Deng et al., 2024; Sabogal-Guaqueta et al., 2024; Wang et al., 2024). Recently developed microglia-containing organoids exhibit stimulus-specific functional responses and enable the evaluation of complement and TREM2-targeted therapeutic interventions within human genetic contexts, thereby directly probing the contribution of neuroinflammation to sevoflurane-associated phenotypes (Buonfiglioli et al., 2025; Sabogal-Guaqueta et al., 2024).

Multimodal and longitudinal functional assessment should become the standard in organoid research (Cao, 2022; Li et al., 2023). High-density microelectrode arrays (MEAs), non-invasive long-term biosensors, and soft mesh-based bioelectronics now enable continuous monitoring of oscillatory coupling and neuronal synchrony over months without requiring destructive endpoints, thereby linking transient instabilities in progenitor zones to persistent network-level “scars” (Giandomenico et al., 2020; Jin et al., 2025; Li et al., 2022). Integrating these electrophysiological recordings with single-cell multi-omics profiling establishes aligned analytical axes—spanning structural architecture, cellular metabolism, and functional electrophysiology—that provide a comprehensive framework for scoring and cross-laboratory comparison of dose-duration-timing matrices in neurotoxicity studies (Cao, 2022; Fan et al., 2022; Hu et al., 2025; Li et al., 2023).

Circuit-level realism necessitates moving beyond single-region models (Velasco et al., 2019; Xiang et al., 2017). Assembloids that integrate cortical regions with interneuron-generating domains, or that connect cortical tissue to BBB-like or thalamic modules, enable direct testing of whether sevoflurane disrupts critical developmental processes such as long-range projection timing, interneuron migration, and cross-regional synchrony—phenomena that remain inaccessible in isolated, monotypic tissues (Dao et al., 2024; Velasco et al., 2019; Xiang et al., 2017). Concurrently, *in vivo* anchoring through transplantation of vascularized organoids provides a physiological benchmark for “normal maturation” within perfused and innervated host environments, thereby improving the calibration of effect sizes observed *in vitro* (Mansour et al., 2018).

Finally, exposure paradigms must be precisely aligned with developmental milestones and incorporate recovery periods (Giandomenico et al., 2020; Li et al., 2023). Maternal exposure studies and human organoid models consistently demonstrate that sevoflurane can transiently disrupt interkinetic nuclear migration (INM) and accelerate lineage commitment, with only partial functional recovery observed at later stages—indicating a narrow, developmentally sensitive window of reversibility (Jiang et al., 2021; Lee et al., 2022). Future experimental designs should therefore: (i) stage-lock cultures prior to dosing; (ii) include dedicated washout or recovery phases to quantify hysteresis and assess residual effects; and (iii) pre-register mechanistic rescue hypotheses—such as modulation of ferroptosis, mitochondrial stabilization, or inhibition of complement/TREM2 signaling—using composite endpoints that integrate progenitor dynamics, network synchrony, and behaviorally anchored surrogates wherever feasible (Deng et al., 2024; Kang et al., 2023; Sun M. et al., 2021; Wang et al., 2024).

### 6.2 Challenges and future prospects in sevoflurane neurotoxicity research

#### 6.2.1 Bridging the translational gap

Human brain development diverges from that of rodents in terms of developmental tempo, cellular composition, and gene regulatory networks, rendering direct extrapolation from animal models inherently uncertain (Li et al., 2023; Qian et al., 2019; Trujillo and Muotri, 2018). Brain organoids and related iPSC-derived platforms help bridge this species gap by recapitulating human-specific developmental milestones—such as radial glial dynamics and interneuron migration—in three-dimensional architectures (Lancaster and Knoblich, 2014; Velasco et al., 2019; Xiang et al., 2017). This enables anesthetic effects to be directly mapped onto native human neurodevelopmental trajectories, rather than inferred through cross-species translation (Fan et al., 2022; Hu et al., 2025; Li et al., 2023). For further discussion, see a dedicated review demonstrating that 3D human brain organoids exhibit greater alignment with clinical neurotoxicity phenotypes than either 2D cultures or rodent models (Bai, 2024; Cao, 2022).

#### 6.2.2 Methodological “three hard problems”: maturity, heterogeneity, and microenvironment

Current organoid models face three major challenges: (i) batch-to-batch and intra-organoid variability that alters progenitor cell proportions; (ii) insufficient vascular and immune niches, which lead to hypoxia and stress-related artifacts in long-term cultures; and (iii) incomplete recapitulation of inter-regional neural circuitry (Gong et al., 2021; Li et al., 2023). Promising solutions now emerging include endothelial co-culture and microfluidic perfusion—enabling increased tissue viability and reduced necrotic core formation—routine integration of iPSC-derived microglia to model complement/TREM2-mediated synaptic pruning, and the use of assembloids to reconstruct long-range projections and inhibitory interneuron migration (Dao et al., 2024; Li et al., 2022; Sabogal-Guaqueta et al., 2024). A recent perspective further advocates for human iPSC-derived organoids, assembloids, and blood-brain barrier (BBB) modules as physiologically relevant platforms to investigate mechanisms of alcohol- and anesthetic-induced brain injury in human-specific contexts (Bai, 2024).

#### 6.2.3 Defining exposure paradigms with clinical relevance

Sevoflurane exerts non-binary effects that are modulated by concentration, duration, developmental stage, and metabolic context—including oxygen and glucose availability (Neag et al., 2020; Patel and Goa, 1996; Sun M. et al., 2021). Organoids enable the implementation of programmable “dose-duration-timing” matrices precisely aligned to neurodevelopmentally defined windows (fetal-like vs. infant-like), facilitating direct, controlled comparisons between brief induction-like, prolonged maintenance-like, and recurrent exposures (Giandomenico et al., 2020; Hu et al., 2025; Lee et al., 2022; Li et al., 2023). Outcomes can be systematically tracked across multiple levels: morphological (ventricular and subventricular zone thickness; Ki67/PH3 labeling), dynamic (interkinetic nuclear migration coherence), and functional (multi-electrode array recordings, long-term biosensing) (Giandomenico et al., 2020; Jin et al., 2025; Lee et al., 2022; Li et al., 2023).

#### 6.2.4 What organoids reveal about developmentally sensitive windows

Maternal exposure studies and organoid models demonstrate transient disruption of interkinetic nuclear migration (INM) and accelerated lineage commitment, with only partial functional recovery—providing evidence for narrow, stage-specific windows of reversibility (Jiang et al., 2021; Lee et al., 2022; Oyefeso et al., 2021; Shang et al., 2022). Midbrain organoids further exhibit reduced proliferation and premature dopaminergic differentiation following prolonged sevoflurane exposure, whereas shorter exposures markedly attenuate these effects—highlighting a duration-dependent neurodevelopmental vulnerability that mirrors clinical perioperative exposure patterns (Oyefeso et al., 2021; Shang et al., 2022).

#### 6.2.5 Toward quantitative and reproducible risk profiles

Future efforts should prioritize the following: (i) standardized minimum reporting criteria, including cell line provenance and karyotype, batch identity, exposure staging, real-time monitoring of dissolved O2 and glucose levels, and statistical power; (ii) developmental stage-locking prior to compound administration, combined with a defined washout period to assess potential hysteresis (temporal lag) effects; (iii) neuroimmune-competent models that routinely incorporate iPSC-derived microglia and systematically evaluate the complement-TREM2 signaling axis; (iv) vascularized and blood-brain barrier (BBB)-integrated assembloids to enhance pharmacokinetic/pharmacodynamic relevance and translatability; and (v) streamlined, nonredundant multimodal outcome scoring that integrates markers of oxidative stress and ferroptosis, mitochondrial homeostasis, and network-level synchrony—enabling the generation of comparable “injury fingerprints” for cross-laboratory validation and benchmarking (Li et al., 2023; Sabogal-Guaqueta et al., 2024; Sun M. et al., 2021; Zhao and Haddad, 2024).

#### 6.2.6 Clinical relevance and translational implications

Brain organoids provide a human-relevant bridge between laboratory-level mechanistic signals and clinically interpretable outcomes in assessing the potential developmental neurotoxicity of sevoflurane (Fan et al., 2022; Li et al., 2023; Qian et al., 2019; Trujillo and Muotri, 2018). Compared with rodent or nonhuman primate models, region-specific human iPSC or ESC-derived organoids more faithfully recapitulate the lineage trajectories and laminar organization of the developing human brain, enabling direct observation—within a human developmental context—of how sevoflurane disrupts the tempo of neurogenesis and how these disruptions are modulated by dose and duration (Lancaster and Knoblich, 2014; Lancaster et al., 2013; Velasco et al., 2019; Xiang et al., 2017). In midbrain organoids, prolonged exposure to 2% sevoflurane suppresses progenitor cell proliferation, increases apoptosis, and promotes premature dopaminergic lineage differentiation at the transcriptomic level; in contrast, shorter exposures elicit markedly weaker transcriptional changes, indicating that an “exposure-duration threshold” is a critical determinant of developmentally relevant outcomes and aligning experimental designs with the principle of cumulative perioperative anesthetic exposure in clinical practice (Shang et al., 2022).

At the cortical level, maternal sevoflurane exposure transiently disrupts interkinetic nuclear migration (INM) in radial glial progenitors—a phenotype that is faithfully recapitulated in human 3D cerebral organoids. Activation of the Notch signaling pathway through Jag1 or NICD rescues this defect, indicating that the Notch-INM axis may represent a mechanistically grounded and actionable target for interventions during embryonic anesthesia (Jiang et al., 2021). Notably, this cellular and developmental perturbation does not lead to significant spatial learning or memory deficits in offspring during young adulthood, which is broadly consistent with clinical observations that “single short exposures are generally safe,” suggesting potential developmental compensation or delayed phenotypes; therefore, extended and granular neuropsychological follow-up is warranted (Qiu et al., 2024; Warner et al., 2018; Xiao et al., 2022). Furthermore, multiple studies report that maternal sevoflurane exposure can induce aberrant fetal prefrontal cortical development accompanied by cognitive impairments in offspring, providing experimental support for potential long-term neurobehavioral risks following prenatal anesthetic exposure (Song et al., 2017).

Mechanistic reviews integrate multiple sevoflurane-associated developmental pathways—neuronal injury and apoptosis, impaired synapse and circuit formation, Tau phosphorylation, mitochondrial dysfunction-inflammation-IL-6 signaling, and non-coding RNA regulation—and converge on a dose-duration-pathway view: brief or single exposures have minimal long-term cognitive consequences, whereas prolonged or repeated exposures are associated with greater neurodevelopmental risk (Sun M. et al., 2021). This dose-duration-pathway framework provides a structured approach for translating organoid-derived findings into clinically relevant endpoints and identifies candidate neuroprotective strategies, including anti-inflammatory interventions, mitochondrial stabilization, and modulation of the Tau signaling axis (Sun M. et al., 2021).

Recent clinical evidence helps calibrate the translational gap between organoid-derived molecular and cellular signals and real-world neurodevelopmental outcomes (Warner et al., 2018; Xiao et al., 2022). Systematic reviews and landmark trials (e.g., GAS, PANDA, MASK) indicate that the majority of data show no significant reduction in global IQ or core cognitive function following a single, brief episode of general anesthesia in early life. In contrast, multiple or longer cumulative exposures are more consistently associated with subtle alterations in specific cognitive and behavioral subdomains—a finding that closely aligns with the organoid-based pattern of “stronger effects with prolonged exposure, minimal effects with brief exposure.” Collectively, the balanced statement is that a single short exposure does not, on average, impair global intelligence, whereas repeated or extended exposures may increase risk for domain-specific neurocognitive changes (Warner et al., 2018; Xiao et al., 2022).

For model-to-bedside translation, three priorities stand out (Warner et al., 2018; Xiao et al., 2022). First, clinical alignment of exposure metrics: explicitly translate clinical parameters—such as MAC⋅hours, number of exposures, and inter-exposure intervals—into organoid experimental designs, and incorporate perioperative covariates (e.g., hypotension, hypoperfusion, inflammation/infection, hypoxemia) to more accurately reflect real-world anesthetic contexts (Patel and Goa, 1996; Sun M. et al., 2021; Warner et al., 2018; Xiao et al., 2022). This integration helps narrow the gap between robust experimental effects observed *in vitro* and the relatively modest clinical outcomes reported in population studies (Warner et al., 2018; Xiao et al., 2022). Second, biomarker-endpoint bridging: for organoid-validated pathways with strong mechanistic support (e.g., Notch-INM, mitochondrial dysfunction/IL-6 signaling, Tau phosphorylation), collect early postoperative biofluid biomarkers and integrate them with longitudinal neuropsychological assessments to develop predictive models that link acute biological perturbations to delayed functional outcomes (Sun M. et al., 2021; Warner et al., 2018; Xiao et al., 2022). Third, risk stratification: embed prospective follow-up or interventional studies within cohorts at higher developmental risk—such as preterm infants, genetically susceptible children, or those undergoing multiple anesthetic exposures—to test organoid-identified druggable pathways as potential targets for perioperative neuroprotection (Sun M. et al., 2021; Warner et al., 2018; Xiao et al., 2022). Field commentaries increasingly advocate a shift from exploratory animal studies and broad population surveys toward a structured mechanism-biomarker-trial pipeline—a niche where organoids provide outsized value (Sun M. et al., 2021; Xiao et al., 2022).

Constraints of organoids must be acknowledged: absence of vasculature and immune niches, size limits with necrotic cores, and incomplete pharmacokinetic/pharmacodynamic (PK/PD) reconstruction can blunt ecological validity (Dao et al., 2024; Giandomenico et al., 2020; Li et al., 2023; Zhao and Haddad, 2024). To enhance relevance, platforms should incorporate functional microvasculature/endothelium and microglia, add physiologic shear and biomaterial scaffolds, and couple with BBB co-culture; where appropriate, transplantation or fusion strategies can lift system-level fidelity (Dao et al., 2024; Li et al., 2022; Mansour et al., 2018; Sabogal-Guaqueta et al., 2024). These enhancements enable evaluation of sevoflurane’s effects within an integrated development-hemodynamics-inflammation frame that better reflects *in vivo* complexity (Dao et al., 2024; Sabogal-Guaqueta et al., 2024). Given the consistent pattern—minimal impact after single/brief exposures but greater concern with prolonged/repeated exposures—perioperative teams should prioritize essential surgery without delay, minimize anesthesia duration/repetition, optimize intraoperative physiology/inflammation, and establish structured long-term follow-up for higher-risk children (Sun M. et al., 2021; Warner et al., 2018).

## 7 Conclusion

In summary, organoids map potential sevoflurane effects onto the human neurodevelopmental timeline and align directionally with clinical evidence: mechanistic signals are robust, whereas clinical risk depends on exposure parameters and population context. The next step is to convert reproducible organoid pathway readouts into measurable clinical biomarkers and actionable targets, and to close the loop with longitudinal cohorts plus embedded trials—achieving precise model-to-bedside extrapolation.

## References

[B1] Bai X. (2024). Use of induced pluripotent stem cell-derived brain cells, organoids, assembloids, and blood-brain barrier models in understanding alcohol and anesthetic-induced brain injuries: An emerging perspective. *Neural Regen. Res.* 19 953–954. 10.4103/1673-5374.385297 37862185 PMC10749634

[B2] Briner A. De Roo M. Dayer A. Muller D. Habre W. Vutskits L. (2010). Volatile anesthetics rapidly increase dendritic spine density in the rat medial prefrontal cortex during synaptogenesis. *Anesthesiology* 112 546–556. 10.1097/ALN.0b013e3181cd7942 20124985

[B3] Buonfiglioli A. Kübler R. Missall R. De Jong R. Chan S. Haage V. et al. (2025). A microglia-containing cerebral organoid model to study early life immune challenges. *Brain Behav. Immun.* 123 1127–1146. 10.1016/j.bbi.2024.11.008 39500415 PMC11753195

[B4] Cao Y. (2022). The uses of 3D human brain organoids for neurotoxicity evaluations: A review. *Neurotoxicology* 91 84–93. 10.1016/j.neuro.2022.05.004 35561940

[B5] Cornelissen L. Kim S. Lee J. Brown E. Purdon P. Berde C. (2018). Electroencephalographic markers of brain development during sevoflurane anaesthesia in children up to 3 years old. *Br. J. Anaesth.* 120 1274–1286. 10.1016/j.bja.2018.01.037 29793594 PMC6617966

[B6] Dai J. Li X. Wang C. Gu S. Dai L. Zhang J. et al. (2021). Repeated neonatal sevoflurane induced neurocognitive impairment through NF-κB-mediated pyroptosis. *J. Neuroinflammation* 18:180. 10.1186/s12974-021-02233-9 34419096 PMC8380327

[B7] Dao L. You Z. Lu L. Xu T. Sarkar A. Zhu H. et al. (2024). Modeling blood-brain barrier formation and cerebral cavernous malformations in human PSC-derived organoids. *Cell. Stem. Cell.* 31 818–833.e11. 10.1016/j.stem.2024.04.019 38754427 PMC11162335

[B8] Deng L. Song S. Zhao W. Meng X. Liu H. Zheng Q. et al. (2024). Triggering receptor expressed on myeloid cells 2 alleviated sevoflurane-induced developmental neurotoxicity via microglial pruning of dendritic spines in the CA1 region of the hippocampus. *Neurosci. Bull.* 40 1215–1229. 10.1007/s12264-024-01260-9 39078595 PMC11365924

[B9] Dixon T. Muotri A. (2023). Advancing preclinical models of psychiatric disorders with human brain organoid cultures. *Mol. Psychiatry* 28 83–95. 10.1038/s41380-022-01708-2 35948659 PMC9812789

[B10] Fan P. Wang Y. Xu M. Han X. Liu Y. (2022). The application of brain organoids in assessing neural toxicity. *Front. Mol. Neurosci.* 15:799397. 10.3389/fnmol.2022.799397 35221913 PMC8864968

[B11] Gao T. Huang Z. (2024). Novel insights into sevoflurane-induced developmental neurotoxicity mechanisms. *Epigenomics* 16 1231–1252. 10.1080/17501911.2024.2395250 39316776 PMC11485883

[B12] Giandomenico S. Sutcliffe M. Lancaster M. (2020). Generation and long-term culture of advanced cerebral organoids for studying later stages of neural development. *Nat. Protoc.* 16 579–602. 10.1038/s41596-020-00433-w 33328611 PMC7611064

[B13] Gong J. Meng T. Yang J. Hu N. Zhao H. Tian T. (2021). Three-dimensional in vitro tissue culture models of brain organoids. *Exp. Neurol.* 339:113619. 10.1016/j.expneurol.2021.113619 33497645

[B14] Hu D. Cao Y. Cai C. Wang G. Zhou M. Peng L. et al. (2025). Establishment of human cerebral organoid systems to model early neural development and assess the central neurotoxicity of environmental toxins. *Neural Regen. Res.* 20 242–252. 10.4103/NRR.NRR-D-23-00928 38767489 PMC11246146

[B15] Huang J. Zhu Y. Tang J. Liu Y. Lu M. Zhang R. et al. (2025). Navigating brain organoid maturation: From benchmarking frameworks to multimodal bioengineering strategies. *Biomolecules* 15:1118. 10.3390/biom15081118 40867563 PMC12383812

[B16] Huang Y. Guo L. Cao C. Ma R. Huang Y. Zhong K. et al. (2022). Silver nanoparticles exposure induces developmental neurotoxicity in hiPSC-derived cerebral organoids. *Sci. Total Environ.* 845:157047. 10.1016/j.scitotenv.2022.157047 35780879

[B17] Jiang M. Tang T. Liang X. Li J. Qiu Y. Liu S. et al. (2021). Maternal sevoflurane exposure induces temporary defects in interkinetic nuclear migration of radial glial progenitors in the fetal cerebral cortex through the Notch signalling pathway. *Cell. Prolif.* 54:e13042. 10.1111/cpr.13042 33955094 PMC8168415

[B18] Jin Y. Guo Y. Li Q. Wu L. Ge Y. Zhao J. (2025). Non-invasive and long-term electrophysiological monitoring sensors for cerebral organoids differentiation. *Biosensors* 15:173. 10.3390/bios15030173 40136970 PMC11940203

[B19] Kang L. Piao M. Liu N. Gu W. Feng C. (2023). Sevoflurane exposure induces neuronal cell ferroptosis initiated by increase of intracellular hydrogen peroxide in the developing brain via ER stress ATF3 activation. *Mol. Neurobiol.* 61 2313–2335. 10.1007/s12035-023-03695-z 37874483 PMC10972952

[B20] Lancaster M. Knoblich J. (2014). Organogenesis in a dish: Modeling development and disease using organoid technologies. *Science* 345:1247125. 10.1126/science.1247125 25035496

[B21] Lancaster M. Renner M. Martin C. Wenzel D. Bicknell L. Hurles M. et al. (2013). Cerebral organoids model human brain development and microcephaly. *Nature* 501 373–379. 10.1038/nature12517 23995685 PMC3817409

[B22] Lee J. Bae D. Choi W. Cho C. Bang Y. Yoo J. (2022). Effects of sevoflurane exposure on fetal brain development using cerebral organoids. *J. Mol. Neurosci.* 72 2440–2450. 10.1007/s12031-022-02080-0 36478139

[B23] Li T. Liu Y. Forro C. Yang X. Beker L. Bao Z. et al. (2022). Stretchable mesh microelectronics for the biointegration and stimulation of human neural organoids. *Biomaterials* 290:121825. 10.1016/j.biomaterials.2022.121825 36326509 PMC9879137

[B24] Li Y. Zeng P. Wu J. Luo Z. (2023). Advances and applications of brain organoids. *Neurosci. Bull.* 39 1703–1716. 10.1007/s12264-023-01065-2 37222855 PMC10603019

[B25] Liang F. Li M. Xu M. Zhang Y. Dong Y. Soriano S. et al. (2023). Sevoflurane anaesthesia induces cognitive impairment in young mice through sequential tau phosphorylation. *Br. J. Anaesth.* 131 726–738. 10.1016/j.bja.2023.06.059 37537117 PMC10541551

[B26] Liu J. Li L. Xie P. Zhao X. Shi D. Zhang Y. et al. (2022). Sevoflurane induced neurotoxicity in neonatal mice links to a GSK3β/Drp1-dependent mitochondrial fission and apoptosis. *Free Radic. Biol. Med.* 181 72–81. 10.1016/j.freeradbiomed.2022.01.031 35122996

[B27] Lu P. Liang F. Dong Y. Xie Z. Zhang Y. (2023). Sevoflurane induces a cyclophilin D-dependent decrease of neural progenitor cells migration. *Int. J. Mol. Sci.* 24:6746. 10.3390/ijms24076746 37047719 PMC10095407

[B28] Mansour A. Gonçalves J. Bloyd C. Li H. Fernandes S. Quang D. et al. (2018). An in vivo model of functional and vascularized human brain organoids. *Nat. Biotechnol.* 36 432–441. 10.1038/nbt.4127 29658944 PMC6331203

[B29] Miao M. Han Y. Wang Y. Wang J. Zhu R. Yang Y. et al. (2024). Dysregulation of iron homeostasis and ferroptosis in sevoflurane and isoflurane associated perioperative neurocognitive disorders. *CNS Neurosci. Ther.* 30:e14553. 10.1111/cns.14553 38334231 PMC10853900

[B30] Neag M. Mitre A. Catinean A. Mitre C. (2020). An overview on the mechanisms of neuroprotection and neurotoxicity of isoflurane and sevoflurane in experimental studies. *Brain Res. Bull.* 165 281–289. 10.1016/j.brainresbull.2020.10.011 33080307

[B31] Nguyen H. (2022). Generation of iPSC-derived brain organoids for drug testing and toxicological evaluation. *Methods Mol. Biol.* 2474 93–105. 10.1007/978-1-0716-2213-1_10 35294759

[B32] Oyefeso F. Muotri A. Wilson C. Pecaut M. (2021). Brain organoids: A promising model to assess oxidative stress-induced central nervous system damage. *Dev. Neurobiol.* 81 653–670. 10.1002/dneu.22828 33942547 PMC8364474

[B33] Park S. Sun W. (2025). Toxicity assessment using neural organoids: Innovative approaches and challenges. *Toxicol. Res.* 41 91–103. 10.1007/s43188-025-00279-y 40013084 PMC11850696

[B34] Patel S. Goa K. (1996). Sevoflurane. A review of its pharmacodynamic and pharmacokinetic properties and its clinical use in general anaesthesia. *Drugs* 51 658–700. 10.2165/00003495-199651040-00009 8706599

[B35] Qian X. Song H. Ming G. (2019). Brain organoids: Advances, applications and challenges. *Development* 146:dev166074. 10.1242/dev.166074 30992274 PMC6503989

[B36] Qiu L. Li H. Li B. Ek J. Zhang X. Chen Y. et al. (2024). Sevoflurane exposure in early life: Mitochondrial dysfunction and neurotoxicity in immature rat brains without long-term memory loss. *Sci. Rep.* 14:28747. 10.1038/s41598-024-79150-3 39567567 PMC11579499

[B37] Qiu L. Zhu C. Bodogan T. Gómez-Galán M. Zhang Y. Zhou K. et al. (2016). Acute and long-term effects of brief sevoflurane anesthesia during the early postnatal period in rats. *Toxicol. Sci.* 149 121–133. 10.1093/toxsci/kfv219 26424773

[B38] Sabogal-Guaqueta A. Mitchell-Garcia T. Hunneman J. Voshart D. Thiruvalluvan A. Foijer F. et al. (2024). Brain organoid models for studying the function of iPSC-derived microglia in neurodegeneration and brain tumours. *Neurobiol. Dis.* 203:106742. 10.1016/j.nbd.2024.106742 39581553

[B39] Sandoval S. Cappuccio G. Kruth K. Osenberg S. Khalil S. Méndez-Albelo N. et al. (2024). Rigor and reproducibility in human brain organoid research: Where we are and where we need to go. *Stem Cell Reports* 19 796–816. 10.1016/j.stemcr.2024.04.008 38759644 PMC11297560

[B40] Shang J. Li B. Fan H. Liu P. Zhao W. Chen T. et al. (2022). Sevoflurane promotes premature differentiation of dopaminergic neurons in hiPSC-derived midbrain organoids. *Front. Cell. Dev. Biol.* 10:941984. 10.3389/fcell.2022.941984 36176283 PMC9513420

[B41] Shen X. Dong Y. Xu Z. Wang H. Miao C. Soriano S. et al. (2013). Selective anesthesia-induced neuroinflammation in developing mouse brain and cognitive impairment. *Anesthesiology* 118 502–515. 10.1097/ALN.0b013e3182834d77 23314110 PMC3580002

[B42] Song R. Ling X. Peng M. Xue Z. Cang J. Fang F. (2017). Maternal sevoflurane exposure causes abnormal development of fetal prefrontal cortex and induces cognitive dysfunction in offspring. *Stem. Cells Int.* 2017:6158468. 10.1155/2017/6158468 29098009 PMC5643154

[B43] Sun L. Li Y. Wang D. Hong X. (2024). SESN2 attenuates sevoflurane-induced cognitive impairment and neuroinflammation in rats. *Exp. Brain Res.* 242 375–384. 10.1007/s00221-023-06757-9 38129329

[B44] Sun M. Xie Z. Zhang J. Leng Y. (2021). Mechanistic insight into sevoflurane-associated developmental neurotoxicity. *Cell. Biol. Toxicol.* 38 927–943. 10.1007/s10565-021-09677-y 34766256 PMC9750936

[B45] Sun N. Meng X. Liu Y. Song D. Jiang C. Cai J. (2021). Applications of brain organoids in neurodevelopment and neurological diseases. *J. Biomed. Sci.* 28:30. 10.1186/s12929-021-00728-4 33888112 PMC8063318

[B46] Trujillo C. Muotri A. (2018). Brain organoids and the study of neurodevelopment. *Trends Mol. Med.* 24 982–990. 10.1016/j.molmed.2018.09.005 30377071 PMC6289846

[B47] Useinovic N. Near M. Cabrera O. Boscolo A. Milosevic A. Harvey R. et al. (2023). Neonatal sevoflurane exposure induces long-term changes in dendritic morphology in juvenile rats and mice. *Exp. Biol. Med.* 248 641–655. 10.1177/15353702231170003 37309741 PMC10350807

[B48] Velasco S. Kedaigle A. Simmons S. Nash A. Rocha M. Quadrato G. et al. (2019). Individual brain organoids reproducibly form cell diversity of the human cerebral cortex. *Nature* 570 523–527. 10.1038/s41586-019-1289-x 31168097 PMC6906116

[B49] Wan Y. Wu Z. Li X. Zhao P. (2022). Maternal sevoflurane exposure induces neurotoxicity in offspring rats via the CB1R/CDK5/p-tau pathway. *Front. Pharmacol.* 13:1066713. 10.3389/fphar.2022.1066713 36703741 PMC9871255

[B50] Wang G. Liu H. Meng X. Chen Y. Zhao W. Li W. et al. (2024). Complement C1q-mediated microglial synaptic elimination by enhancing desialylation underlies sevoflurane-induced developmental neurotoxicity. *Cell. Biosci.* 14:42. 10.1186/s13578-024-01223-7 38556890 PMC10983687

[B51] Warner D. Shi Y. Flick R. (2018). Anesthesia and neurodevelopment in children: Perhaps the end of the beginning. *Anesthesiology* 128 700–703. 10.1097/ALN.0000000000002121 29533967 PMC5854201

[B52] Xiang Y. Tanaka Y. Patterson B. Kang Y. Govindaiah G. Roselaar N. et al. (2017). Fusion of regionally specified hPSC-derived organoids models human brain development and interneuron migration. *Cell. Stem. Cell.* 21 383–398.e7. 10.1016/j.stem.2017.07.007 28757360 PMC5720381

[B53] Xiao A. Feng Y. Yu S. Xu C. Chen J. Wang T. et al. (2022). General anesthesia in children and long-term neurodevelopmental deficits: A systematic review. *Front. Mol. Neurosci.* 15:972025. 10.3389/fnmol.2022.972025 36238262 PMC9551616

[B54] Xu F. Han L. Wang Y. Deng D. Ding Y. Zhao S. et al. (2023). Prolonged anesthesia induces neuroinflammation and complement-mediated microglial synaptic elimination involved in neurocognitive dysfunction and anxiety-like behaviors. *BMC Med.* 21:7. 10.1186/s12916-022-02705-6 36600274 PMC9814183

[B55] Yang L. Ton H. Zhao R. Geron E. Li M. Dong Y. et al. (2020). Sevoflurane induces neuronal activation and behavioral hyperactivity in young mice. *Sci. Rep.* 10:11226. 10.1038/s41598-020-66959-x 32641746 PMC7343864

[B56] Zeng F. Zhou M. Li Q. Hu H. Chen C. (2024). Sevoflurane promotes neuronal ferroptosis via upregulation of PLIN4 to modulate the hippo signaling pathway. *Neurotoxicology* 105 1–9. 10.1016/j.neuro.2024.08.001 39182851

[B57] Zhang Q. Li Y. Wang X. Yin C. Zhou Q. Guo J. et al. (2022). Sevoflurane exposure causes neuronal apoptosis and cognitive dysfunction by inducing ER stress via activation of the inositol 1, 4, 5-trisphosphate receptor. *Front. Aging Neurosci.* 14:990679. 10.3389/fnagi.2022.990679 36337694 PMC9631943

[B58] Zhang Y. Tang C. He Y. Zhang Y. Li Q. Zhang T. et al. (2024). Semaglutide ameliorates Alzheimer’s disease and restores oxytocin in APP/PS1 mice and human brain organoid models. *Biomed. Pharmacother.* 180:117540. 10.1016/j.biopha.2024.117540 39405916

[B59] Zhao H. Haddad G. (2024). Brain organoid protocols and limitations. *Front. Cell. Neurosci.* 18:1351734. 10.3389/fncel.2024.1351734 38572070 PMC10987830

[B60] Zhao X. Shi D. Du Y. Hu C. Li Y. Liu J. et al. (2022). Repeated neonatal exposure to sevoflurane induces age-dependent impairments in cognition and synaptic plasticity in mice. *Neurosci.* 44 153–161. 10.1159/000523730 35203077

[B61] Zheng H. Dong Y. Xu Z. Crosby G. Culley D. Zhang Y. et al. (2013). Sevoflurane anesthesia in pregnant mice induces neurotoxicity in fetal and offspring mice. *Anesthesiology* 118 516–526. 10.1097/ALN.0b013e3182834d5d 23314109 PMC3580035

